# Delayed Intraventricular Hemorrhage following a Ventriculoperitoneal Shunt Placement: Exploring the Surgical Anatomy of a Rare Complication

**DOI:** 10.1155/2017/3953248

**Published:** 2017-11-08

**Authors:** Ioannis N. Mavridis, Athanassios Mitropoulos, Constantinos Mantas, Aikaterini Karagianni, Konstantinos Vlachos

**Affiliations:** Department of Neurosurgery, “K.A.T.-N.R.C.” General Hospital of Attica, Nikis Street 2, Kifissia, 14561 Athens, Greece

## Abstract

Ventriculoperitoneal shunt (VPS) placement is one of the commoner neurosurgical procedures worldwide. The purpose of this article is to report a case of delayed intraventricular hemorrhage (IVH) following a VPS and to review the literature regarding anatomic factors that could potentially explain this rare complication. A 78-year-old man with normal pressure hydrocephalus, who underwent an uneventful right VPS placement, suffered from a catastrophic isolated IVH five days later. The reported cases of delayed intracerebral hemorrhage (ICH) following VPS are rare and those with IVH are even rarer. Potential factors of surgical anatomy that could cause delayed ICH/IVH following a VPS procedure include erosion of vasculature by catheter cannulation, multiple attempts at perforation, puncture of the choroid plexus, improper placement of the tubing within the brain parenchyma, VPS system revision, venous infarction, vascular malformations, head trauma, and brain tumors. Other causes include generalized convulsion, VPS system malfunction, increased intracranial or blood pressure, sudden intracranial hypotension, and bleeding disorders. According to the current literature, our case is the first reported delayed isolated IVH after a VPS placement so far. Neurosurgeons should be aware of the delayed ICH/IVH as a rare, potentially fatal complication of VPS, as well as of its risk factors.

## 1. Introduction

Ventriculoperitoneal shunt (VPS) placement is one of the most common surgical procedures done by neurosurgeons worldwide [1]. It is a routine procedure for cerebrospinal fluid (CSF) diversion and is associated with many complications [2, 3], such as shunt malfunction and revision [4], shunt contamination and infection (meningitis) [3, 4], seizures, subdural hemorrhage [3], and intraparenchymal cerebrovascular accidents (infarcts and hemorrhage) [5, 6]. Bleeding into the brain parenchyma or ventricles is an infrequently reported complication in adults who undergo insertion of a VPS [4]. Delayed postoperative intracerebral hemorrhage (ICH) after VPS surgery, at the site of the ventricular catheter [5], is a quite rare and potentially severe event [2, 3].

The purpose of this article is to report a rare case of delayed catastrophic intraventricular hemorrhage (IVH), following a VPS placement for treating normal pressure hydrocephalus (NPH) in an adult male patient, and to review the literature regarding similar cases searching for potential causes. We aimed to explore possible explanations of this rarely described complication with great respect to factors of surgical anatomy which could potentially cause this severe event.

## 2. Materials and Methods

**Figure 1:**
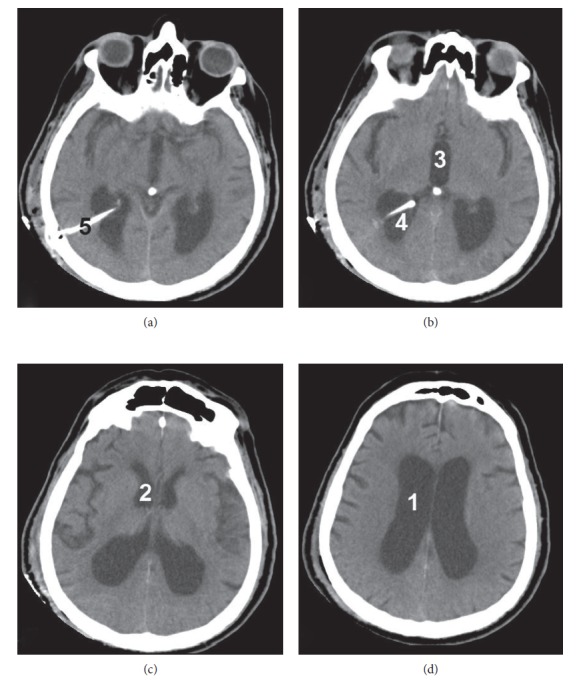
The cerebral CT scan of our patient. (a), (b), (c), (d) Different views (from inferior to superior). Note the large IVH within the right lateral ventricle (1), around the ventricular catheter (2), with extension into the third (3) and left lateral (4) ventricles. The right lateral ventricle is dilated (5), with apparent periventricular edema (6) and midline shift (7).

A 78-year-old male patient diagnosed with NPH was admitted to our department for surgical treatment. His clinical manifestations were gait disturbance, instability, and urinary incontinence. He had a medical history of diabetes mellitus (type 2) difficultly controlled with oral medication, and he was taking acetylsalicylic acid (due to mild peripheral vasculopathy), 100 mg daily, which had been interrupted a week prior to surgery. He underwent an uneventful right VPS placement (fixed pressure valve) under general anesthesia. On postoperative day 1, he was mobilized and showed remarkable clinical improvement. A postoperative cerebral computed tomography (CT) scan, on postoperative day 1, was satisfactory with no findings of intracranial hemorrhage (Figure 1). On postoperative day 2, he was discharged home with instructions to continue antibiotic treatment per os and to start subcutaneous anticoagulant injections (fondaparinux 2.5 mg daily) for 10 days.

**Figure 2:**
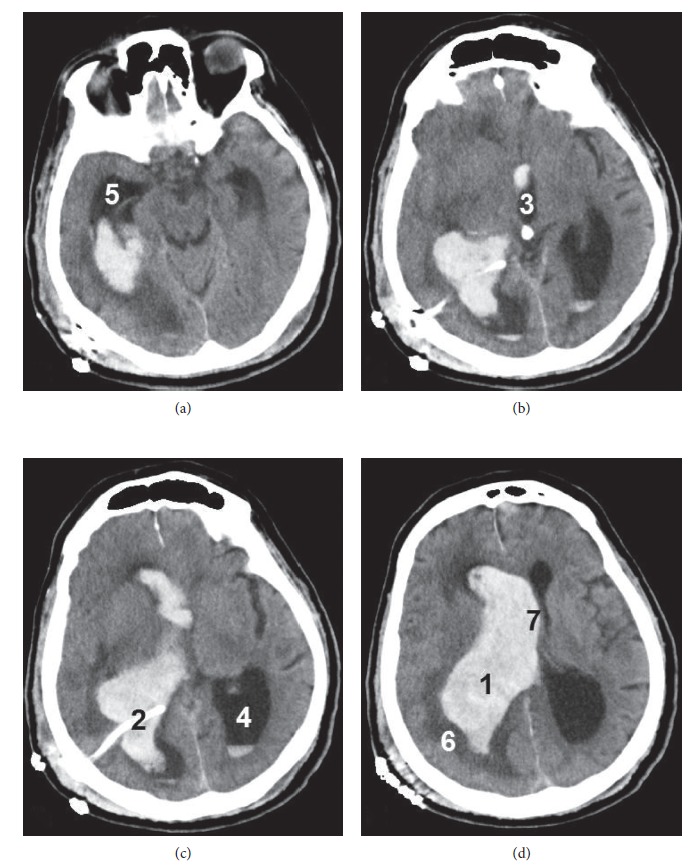
The second postoperative cerebral CT scan of our patient (fifth postoperative day). (a), (b), (c), (d) Different views (from inferior to superior). Note the large IVH within the right lateral ventricle (1), around the ventricular catheter (2), with extension into the third (3) and left lateral (4) ventricles. The right lateral ventricle is dilated (5), with apparent periventricular edema (6) and midline shift (7).

On the fourth postoperative day, the patient started having nonspecific symptoms, and on the fifth postoperative day, he was transferred to the emergency department with right eye deviation, left hemiplegia, and decreased level of consciousness (a Glasgow Coma Scale of 11/15). An urgent CT scan revealed large IVH within the right lateral ventricle with extension into the third and left lateral ventricles. The right lateral ventricle was dilated with periventricular edema and midline shift (Figure 2). The patient was urgently transferred to the operating room, where he underwent a right external ventricular drainage placement (external ventriculostomy) under general anesthesia. He remained intubated, and he was transferred to the intensive care unit for further support and management. He died two weeks later due to sepsis.

## 3. Results and Discussion

### 3.1. Reported Cases

The reported cases of delayed ICH following VPS in the literature are rare. The reported cases of delayed IVH following VPS, however, are even rarer. A PubMed search with the terms “delayed intraventricular hemorrhage” and “ventriculoperitoneal shunt” retrieved only 13 articles. Let us have a closer view of the specific circumstances under which these very uncommon cases occurred.

Kuwamura and Kokunai [7] reported a huge IVH following VPS in a newborn with hydrocephalus. Matsumura et al. [8] reported a serious ICH as a result of measurement for grip power performed on the seventh postoperative day of a VPS. Snow et al. [9] reported a 43-year-old woman with a moderate-sized ICH, which occurred one week after operation for idiopathic hydrocephalus. Umansky et al. [10] reported a case of a 14-month-old boy with a primary intraventricular oligodendroglioma and obstructive hydrocephalus. The child underwent a bilateral VPS and developed a massive and fatal intratumoral hemorrhage initiated by a mild trauma when introducing the left ventricular catheter [10].

Kubokura et al. [11] reported three cases of communicating hydrocephalus after subarachnoid hemorrhage that underwent VPS and suffered from delayed ICH along the ventricular catheter (inserted into the posterior horn of the lateral ventricle). They noted good blood pressure control of the patients postoperatively, no bleeding tendency, and no vascular anomaly in their preoperative angiographies. In the first case, a 60-year-old man, ICH occurred on the eighth (±1) postoperative day as a result of generalized convulsion with coexisting respiratory acidosis. The remaining two cases manifested with headache and hemiparesis suddenly developed immediately after micturition 4 days after operation. In the second case, a 54-year-old man, postoperative CT scan revealed that cranioplasty (performed at the same time as shunt operation) caused mass effect on the shunted side of the brain. In the third case, a 59-year-old woman, ventriculostomy was tried three times, the VPS system was revised, and the revised system did not seem to function well [11].

Additionally, Mascalchi [12] reported a patient with arterial hypertension and a history of cerebrovascular accidents, who underwent VPS for NPH and suffered from a fatal ICH two weeks later [12]. Savitz and Bobroff [4] studied the incidence of delayed ICH (defined as ICH within 48 hours after VPS placement) secondary to ventricular cannulation during shunting procedures, in 125 adult patients with hydrocephalus. They found this rate of delayed ICH (or IVH) after VPS placement (documented by routine postoperative CT scans) to be 4% [4].

Alcázar et al. [6] reported another case of delayed ICH after a VPS procedure. In their case, a right occipital ICH and associated IVH occurred in a 64-year-old woman, six days after the operation for hydrocephalus secondary to subarachnoid hemorrhage [6]. Son and Park [5] reported a 68-year-old woman who underwent VPS through a frontal burr hole and developed hemorrhagic transformation of a massive venous infarction on the second postoperative day [5].

Moreover, Misaki et al. [13] reported four patients who presented with ICH secondary to VPS insertion in order to treat NPH (first patient), hydrocephalus after cerebellar hemorrhage (second patient), and subarachnoid hemorrhage followed by meningitis (third and fourth patients). ICH was confirmed four hours (first patient), two days (second patient), seven days (third patient), and 13 days (fourth patient) after the operation. The first and second patients required intraoperative hemostasis for bleeding from a cortical vein [13].

Another rare case, reported by Khandelwal et al. [1], describes delayed bilateral thalamic bleeding after VPS [1]. Zhou et al. [2] reported two cases of delayed ICH, along the path of the ventricular catheter, which occurred on postoperative days 3 and 5 following VPS [2].

More recently, Okazaki et al. [14] reported two children with late-onset germinal matrix hemorrhage subsequent to VPS insertion for congenital hydrocephalus. Both children were compromised with preceding hypoxic events prior to shunt insertion. The first patient, a female infant with severe craniofacial deformities, was treated with VPS insertion at 35 days of age, and germinal matrix hemorrhage was confirmed one week later. The second patient, a male infant with a large intraparenchymal cyst in the left parietal lobe, underwent VPS at 69 days of age. Postoperative germinal matrix hemorrhage was confirmed, although hydrocephalus was well controlled by VPS insertion. These two cases had also, apart from congenital anomalies in the central nervous system, respiratory problems before shunting [14].

Finally, Ma et al. [3] reported a 67-year-old male patient with a history of head trauma and brain surgery, who underwent a VPS placement for hydrocephalus. The surgery course was uneventful, and no bleeding was revealed in the CT scan after the procedure. However, a massive ICH and IVH occurred 8 hours following adjustment of the valve system on the eighth day after surgery [3].

### 3.2. Possible Causes and Predisposing Factors

According to the above-mentioned cases, potential surgical anatomy-related causes of delayed ICH or IVH following a VPS procedure include erosion of vasculature by catheter cannulation [3, 6, 9], multiple attempts at perforation, puncture of the choroid plexus and improper placement of the tubing within the brain parenchyma [4], VPS system malfunction and revision, cranioplasty performed at the same time as shunt operation [8, 11], vascular malformations, head trauma, brain tumors [6], and venous infarction (caused by coagulation and occlusion of large cortical veins) [5]. To eliminate the risk of postoperative venous infarction, technical precautions to avoid damaging surface vessels in a burr hole are required during VPS [5]. Other potential causes include sudden reduction of CSF pressure after downregulation of the valve [3], increased intracranial or blood pressure, generalized convulsion [8, 11], bleeding disorders [6], and shunt-induced disseminated coagulation profile [1].

According to a suggested theory, small surgical wound in the brain (induced by ventricular catheter insertion) could cause progressive degenerative vascular change and bring about delayed intracranial hemorrhage under some predisposing factors [11]. Furthermore, Misaki et al. [13] defined those happening within two days after VPS as early ICH and those happening between 5 and 13 days after VPS as delayed ICH and attributed bleeding to venous occlusion (due to intraoperative manipulation) in the first group and to the vulnerability of brain tissue induced by a primary brain disease in the second group [13]. Regarding the occurrence of late-onset germinal matrix hemorrhages, after VPS, these may be related to the hypoxic insults on the residual germinal matrix layer and a sudden decrease in CSF pressure [14].

Considering surgical anatomy-related predisposing factors potentially associated with ICH or IVH five days after a VPS placement (as in our case), these may include erosion of vasculature by catheter cannulation [3, 8], vascular malformation, head trauma, and brain tumor [6]. Other predisposing factors include increased intracranial venous pressure produced by Valsalva's effect [9], sudden intracranial hypotension due to downregulation of the valve (when adjusting the valve system) [3], generalized convulsion [11], and bleeding disorder [6].

Table 1 summarizes the reported cases of delayed ICH/IVH (defined as IVH ≥ 5 days) following a VPS placement, so far. According to the current literature, our case is the first reported delayed isolated IVH after a VPS placement, the second case of delayed post-shunting ICH/IVH reported in NPH patients, and the ninth adult case of such a complication (the sixth such a case in male patients), so far (Table 1). Finally, we would like to mention that there is also a case report of delayed ICH that developed in a 7-year-old boy 7 years after VPS surgery due to congenital hydrocephalus, and the authors attributed it to cerebral contusion caused by the ventricular catheter [15].

## 4. Conclusions

In conclusion, neurosurgeons should be aware of the delayed (≥ 5 days postoperatively) ICH/IVH as a rare, potentially fatal complication of VPS. In the absence of other obvious anatomic or pathophysiological predisposing factors, erosion of vasculature during ventricular catheter insertion should be considered a major risk factor of this severe complication. Meticulous attention is therefore mandatory when penetrating the brain parenchyma to reach the lateral ventricle, especially in patients with known predisposing conditions for ICH/IVH. Early postoperative prophylaxis with anticoagulant agents should be avoided or administered with caution in this group of patients.

## Figures and Tables

**Table 1:** The reported cases of delayed ICH/IVH (defined as IVH ≥ 5 days) after VPS placement, so far.

Authors	Year of publication	Age (years)	Gender	Onset (postoperative day)	Isolated IVH
Matusmura et al. [8]	1985	17	Male	7	No
Snow et al. [9]	1986	43	Female	5	No
Mascalchi [12]	1991	68	Male	15	No
Alcázar et al. [6]	2007	64	Female	6	No
Misaki et al. [13]	2010	55	Male	7	No
		64	Male	14	No
Khandelwal et al. [1]	2011	0	—	22	No
Zhou et al. [2]	2012	32	Female	5	No
Okazaki et al. [14]	2013	0	—	7	No
		0	—	7	No
Ma et al. [3]	2015	69	Male	8	No
Our case	2017	78	Male	5	Yes

ICH: intracerebral hemorrhage; IVH: intraventicular hemorrhage.

## Abbreviations

CSF: Cerebrospinal fluid
CT: Computed tomography
ICH: Intracerebral hemorrhage
IVH: Intraventricular hemorrhage
NPH: Normal pressure hydrocephalus
VPS: Ventriculoperitoneal shunt.
